# Childhood individual and family modifiable risk factors for criminal conviction: a 7-year cohort study from Brazil

**DOI:** 10.1038/s41598-022-13975-8

**Published:** 2022-08-04

**Authors:** Carolina Ziebold, Sara Evans-Lacko, Mário César Rezende Andrade, Maurício Scopel Hoffmann, Lais Fonseca, Matheus Ghossain Barbosa, Pedro Mario Pan, Euripedes Miguel, Rodrigo Affonseca Bressan, Luis Augusto Rohde, Giovanni Abrahão Salum, Jair de Jesus Mari, Ary Gadelha

**Affiliations:** 1 Departamento de Psiquiatria, Universidade Federal de São Paulo, Rua Major Maragliano, 241-Vila Mariana, São Paulo, 04017-030 Brazil; 2 Care Policy and Evaluation Centre, London School of Economics and Political Science, London, WC2A 2AE UK; 3 Department of Psychology, Universidade Federal de São João del-Rei, São João del-Rei, Minas Gerais Brazil; 4 Universidade Federal de Santa Maria, Santa Maria, Rio Grande do Sul 97105-900 Brazil; 5 Department of Psychiatry, Universidade Federal Do Rio Grande Do Sul, Porto Alegre, Rio Grande do Sul 90035-003 Brazil; 6 National Institute of Developmental Psychiatry for Children and Adolescents, São Paulo, 05403-010 Brazil; 7 Department of Psychiatry, Universidade de São Paulo, São Paulo, 05403-903 Brazil; 8 ADHD Outpatient and Developmental Psychiatry Programs, Hospital de Clínicas de Porto Alegre, Rio Grande do Sul, 90035-903 Brazil

**Keywords:** Epidemiology, Neuroscience, Psychology, Risk factors

## Abstract

Crime is a major public problem in low- and middle-income countries (LMICs) and its preventive measures could have great social impact. The extent to which multiple modifiable risk factors among children and families influence juvenile criminal conviction in an LMIC remains unexplored; however, it is necessary to identify prevention targets. This study examined the association between 22 modifiable individual and family exposures assessed in childhood (5–14 years, n = 2511) and criminal conviction at a 7-year follow-up (13–21 years, n = 1905, 76% retention rate) in a cohort of young people in Brazil. Population attributable risk fraction (PARF) was computed for significant risk factors. Criminal convictions were reported for 81 (4.3%) youths. Although most children living in poverty did not present criminal conviction (89%), poverty at baseline was the only modifiable risk factor significantly associated with crime (OR 4.14, 99.8% CI 1.38–12.46) with a PARF of 22.5% (95% CI 5.9–36.1%). It suggests that preventing children’s exposure to poverty would reduce nearly a quarter of subsequent criminal convictions. These findings highlight the importance of poverty in criminal conviction, as it includes several deprivations and suggest that poverty eradication interventions during childhood may be crucial for reducing crime among Brazilian youth.

## Introduction

Crimes, such as homicide, robbery, drug trafficking, and violence against others, constitute a major public issue^1^, contributing to substantial health and social costs^2^. Interpersonal violence, for instance, is the fourth leading cause of death globally among young people^3^, and the first among adolescents aged between 15 to 19 years in low- and middle-income countries (LMICs) in Latin America^4^. Crime-related incidents directly impact the life-expectancy of young men living in countries with epidemic rates of violence, such as Brazil and Mexico^5^. Crime impacts the lives of victims^6^ and also measurably impacts the life chances of juvenile offenders, such as through school dropout^7^ and unemployment^8^. Several studies in high-income countries (HICs) predict a reduction in criminal activity through preventive interventions aimed at children^9^ and families^10^. These interventions are supported by longitudinal studies in HICs which provide insights on possible early predictors for later criminal convictions, including family factors such as child maltreatment^11^ and low household income^12^, and individual factors related to externalizing mental health issues, such as conduct problems^13^ and attention deficit/hyperactivity^14^. However, longitudinal investigations regarding modifiable childhood factors associated with juvenile criminal conviction are still limited in LMICs^15^. Moreover, the extent to which multiple modifiable risk factors among children and families influence juvenile criminal conviction in an LMIC remains unexplored; however, it is necessary to identify prevention targets.

A systematic review^15^ found only four longitudinal studies on modifiable childhood risk factors of criminal conviction in LMICs. Main characteristics and results of eight publications derived from these four longitudinal studies are presented in Supplementary Table 1. These studies have provided valuable contributions, including the separated examination of perinatal risk factors (from one^16^ to six^17^ risk factors), sociodemographic exposures at birth (up to five^17^ exposures), and markers of behavioral problems associated to later criminal conviction^18^ or violent crime^19^. Significant findings were not replicated across studies. Unwanted pregnancy was associated with criminal conviction at age of 21–23^16^, but not at older ages^20^ in the 1961–63 Prague Birth Cohort Study, while in the 1993 Pelotas Birth Cohort in Brazil^17^, unwanted pregnancy was only associated with crime among females. Lower household income at birth was the most robust predictor of criminal conviction in the 1982 Pelotas Birth Cohort^21^, but was hardly associated with criminal conviction in the 1993 Birth Cohort^17^. Finally, conduct and hyperactivity problems at the age of 11 years were associated with violent crime in the 1993 Pelotas Birth Cohort^19^ but were not associated with criminal conviction in Mauritius (1969 and 1970 Quatre Bornes and Vacoas Birth cohorts)^18^. Therefore, further research in contemporaneous samples is needed to update the knowledge on the relation between exposures and criminal conviction in LMICs, and to identify a greater number of potential prevention targets in childhood in developing countries, considering the high social burden generated by criminal activities^2^.

Due to the complexity of crime as a construct, the number of factors previously assessed in both HICs and LMICs is limited, and how other perinatal, early child-psychological, educational, and family factors can be predictive of youth criminal conviction remains unanswered. For example, though childhood bullying victimization, parental control, and low academic performance are significantly associated with later antisocial behavior^15^, their relationship with criminal conviction is hardly established. Part of the problem is the inadequate number of studies that investigated the relative influences of multiple modifiable factors associated with criminal conviction within a given population^15^. In the present study was conducted a broader investigation using an “exposure-wide” association approach on multiple modifiable perinatal, individual, family, and school-related exposures associated with youth criminal conviction to identify new potential targets for the prevention of this complex phenomenon. Similar to genome-wide association studies, exposure-wide association studies explore a broad array of potential exposures related to a single outcome (using a hypothesis-free approach)^22^. This epidemiological method has been previously employed to evaluate risk factors for complex phenomena such as depression^23^, cardiovascular diseases, obesity, and household income^24^. To date, no study has used this method to identify modifiable risk factors for criminal conviction. Moreover, when a significant risk factor is identified, the magnitude of its effect on criminal conviction should be explained to inform and guide public measures for crime prevention^25^. However, few studies in criminology have employed the population attributable risk fraction method that could be used by policymakers, professionals, and researchers from different disciplines to estimate the reduction in criminal conviction based on the elimination of a risk factor^26^.

Three other key gaps have been identified. First, the weak association between poverty and crimes that has been reported^15^ may be caused by the sole reliance on income to measure poverty. Indeed, few studies used multidimensional measures of poverty that could capture the diverse vulnerabilities experienced by children living in poverty^27^. The concept of multidimensional childhood poverty, as put forth by the United Nations International Children's Emergency Fund^27^, emphasizes that children living in poverty are exposed to overlapping deprivations other than the lack of income, including limited access to health, housing, nutrition, education, sanitation, water, and other resources; the present study used a proxy of poverty based on lower parental education, diminished purchasing power, housing, and sanitary conditions experienced during childhood. Second, most studies that evaluated childhood externalizing problems as predictors of later criminal convictions used a screening measure of behavioral problems. Such screening measures are used to identify children who are at the risk of being a case of externalizing problems^28^ and have inconsistently shown association with subsequent criminal behaviors^11^. Therefore, the merit of preferring an externalizing psychiatric diagnosis to a behavioral problem measure to recognize early risk of crime involvement remains vague. Third, most of the longitudinal studies in this area do not use appropriate methods to control for potential confounding factors (e.g., incorrectly adjusting for mediators in multivariable models)^15^, or do not perform correction in multiple hypothesis testing. The analyses conducted in the current study were planned to avoid overadjustment (e.g., only non-modifiable variables at the time of exposure were included as controls in multivariable analysis) and multiple tests corrections were considered due to the several potential risk factors in the study.

Based on previous findings and existing research gaps, this study aimed to identify childhood risk factors for criminal conviction at a 7-year follow-up among participants of the Brazilian High-Risk Cohort Study for Psychiatric Disorders (BHRCS)^29^, a school-based cohort of young people living in two large Brazilian cities, São Paulo and Porto Alegre. Sociodemographic, psychological, and family assessments led to the investigation of a panel of 22 potential modifiable perinatal, early life, and childhood risk factors that were associated with later criminal conviction in early adulthood. Specifically, perinatal exposures included unplanned pregnancy, adolescent motherhood (< 18 years), tobacco and alcohol consumption during pregnancy, prematurity, and birthweight. Early life exposures included exclusive breastfeeding duration and childcare attendance. Childhood exposures comprised poverty (including education, housing, sanity, and goods deprivation); contact with father; child and maternal psychiatric diagnosis; family dynamics (cohesion, control, and conflict); maltreatment; bullying; academic performance; school failure, and dropout. Risk factors were analyzed using an exposure-wide association approach and, to show the extent of significant risk factors’ contribution to crime, population attributable risk factions were calculated. This analysis is expected to estimate the proportion of youth criminal convictions which might be potentially avoided through intervention on specifics targets during childhood.

## Results

A total of 1905 participants were interviewed both at baseline (mean age 10.3 years, SD = 1.91, range 5.8–14.4 years) and at the 7-year follow-up (mean age 17.8 years, SD = 1.97, range 13–21 years). Data loss at follow-up were attributed to the following circumstances: site of recruitment (São Paulo), full term pregnancy, no day-care attendance, no contact with biological father, no child or maternal psychiatric diagnosis, and lower age. Supplementary Table 2 shows how differences between the original and final samples were attenuated with inverse probability weights (IPWs).

A total of 81 (4.3%) participants reported some history of criminal conviction at the 7-year follow-up. Information on type of crime was recorded for 41 participants: 34.2% (14/41) theft, 7.3% (3/41) violent robbery, 14.6% (6/41) drug trafficking, and 14.6% (6/41) violent crimes, including one homicide and one attempted homicide. Table 1 presents overall demographic, perinatal, childhood (baseline) clinical, family, and educational characteristics by criminal conviction. Youths with criminal conviction were predominantly male (OR = 3.45, 95% CI [1.77–6.72], *P* < 0.001; approximately 3:1 male/female ratio), but associations were not significant for age (OR = 1.13, 95% CI [0.99–1.29]), non-White ethnicity (OR = 1.46, 95% CI [0.81–2.61]), city (OR = 0.78, 95% CI [0.43–1.42]), and intelligence quotient (IQ) (OR = 0.99, 95% CI [0.97–1.00]). A total of 220 cohort participants were poor at baseline, 11% of them had a criminal conviction at the time of the follow-up.

**Table 1:** Distribution of baseline characteristics by criminal conviction at 7-years follow-up BHRCS.

Characteristics N (%)	Overall		Criminal conviction		No criminal conviction	
Total	1905 (100.0)		81 (4.3)		1824 (95.8)	
**Demographics**						
Age mean (SD)	10.25 (1.91)		10.84 (1.72)		10.22 (1.91)	
Sex male	1033 (54.2)		63 (77.8)		970 (53.2)	
Site Porto Alegre	1002 (52.6)		51 (63.0)		951 (52.1)	
Skin colour White	1162 (61.0)		38 (46.9)		1124 (61.6)	
Black	208 (10.9)		17 (21.0)		191 (10.5)	
Mixed	517 (27.1)		25 (30.9)		492 (27.0)	
Indigenous	9 (0.5)		1 (1.2)		8 (0.4)	
Asian	4 (0.2)		0 (0)		4 (0.2)	
Missing	5 (0.3)		0 (0)		5 (0.3)	
Total Non-white	738 (38.8)		43 (53.1)		695 (38.2)	
**Perinatal characteristics**						
Unplanned pregnancy	1311 (68.8)		62 (76.5)		1249 (68.5)	
Adolescent motherhood	168 (8.8)		14 (17.3)		154 (8.4)	
Smoking during pregnancy	431 (22.6)		19 (23.5)		412 (22.6)	
Alcohol consumption during pregnancy	429 (22.5)		20 (24.7)		409 (22.4)	
Preterm childbirth	293 (15.4)		7 (8.6)		286 (15.7)	
Birth weight Mean (SD)	3214.2 (586.3)		3346.43 (523.9)		3206.0 (588.4)	
**Early childhood**						
Exclusive breastfeeding duration (months)						
Mean (SD)	3.86 (3.32)		4.13 (3.66)		3.85 (3.31)	
No childcare attendance	811 (42.6)		37 (45.7)		774 (42.4)	
*Family characteristics at baseline*						
Poverty	220 (11.6)		24 (29.6)		196 (10.8)	
*Contact with father*						
No	384 (20.2)		23 (28.4)		361 (19.8)	
Deceased	93 (4.9)		7 (8.6)		86 (4.7)	
Maternal psychiatric diagnosis	584 (30.7)		26 (32.1)		558 (30.6)	
Child: Any diagnosis	523 (27.5)		34 (42.0)		483 (26.8)	
Externalizing diagnosis	280 (14.7)		27 (33.3)		253 (13.9)	
Internalizing diagnosis	285 (15.0)		13 (16.1)		272 (14.9)	
Family cohesion Mean (SD)	7.49 (1.90)		7.14 (2.00)		7.51 (1.90)	
Family conflict score Mean (SD)	3.42 (2.24)		4.44 (2.69)		3.37 (2.20)	
Family control score Mean (SD)	4.59 (1.60)		4.32 (1.60)		4.60 (1.60)	
**Child`s characteristics at baseline**						
IQ Mean (SD)	101.38 (17.00)		97.6 (16.68)		101.54 (16.95)	
**Maltreatment exposure**						
High	396 (20.8)		20 (24.7)		376 (20.6)	
**Bullying**						
Victim	435 (22.8)		16 (19.8)		419 (23.0)	
Perpetrator	85 (4.5)		7 (8.6)		78 (4.3)	
Both	218 (11.4)		14 (17.3)		204 (11.2)	
**Academic performance**						
Below average	273 (14.3)		29 (35.8)		244 (13.4)	
Average	1336 (70.1)		48 (59.3)		1228 (70.6)	
Above average	265 (13.9)		4 (4.9)		261 (14.3)	
Missing	31 (1.63)		0 (0.0)		31 (1.7)	
School Failure	374 (19.6)		37 (45.7)		337 (18.5)	
School Dropout	40 (2.1)		2 (2.5)		38 (2.1)	

*IQ* intelligence quotient.

**Figure 1:**
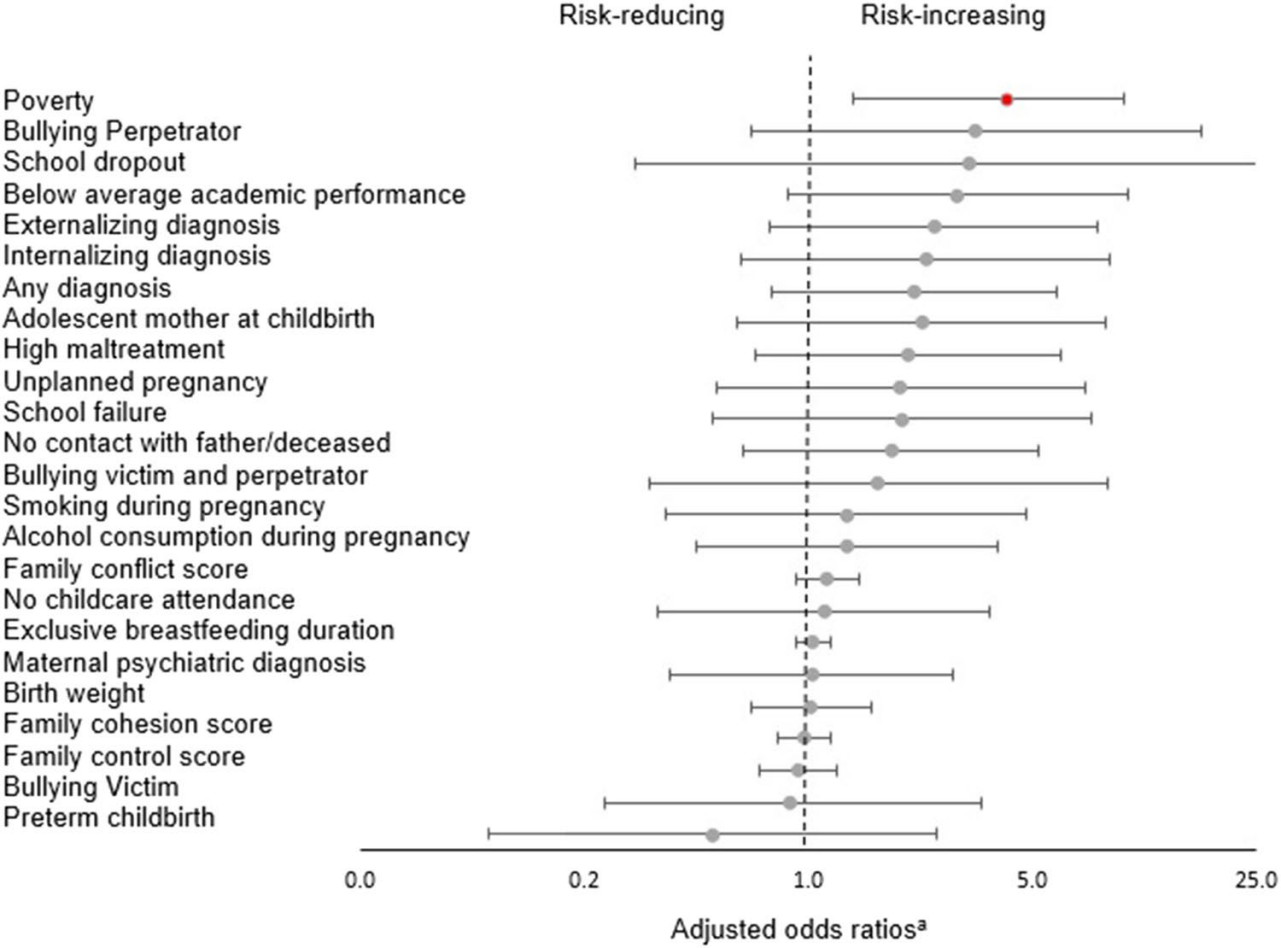
Childhood individual and family modifiable risk factors of criminal conviction, adjusted odds ratios and 99.8% confidence intervals. ^a^Adjusted by sex, age, city, ethnicity, and intelligence quotient. The dash vertical line represents Odds Ratios = 1.00. The red point indicates a significant estimate at *P* < .001.

Table 2 and Fig. 1 present multivariable model results. To minimize the likelihood of type I error, considering that 24 statistical tests were performed, *P* values were adjusted using a conservative Bonferroni-corrected significance threshold (*P* = 0.002). Poverty at baseline was the only modifiable risk factor significantly associated with criminal conviction after 7 years. Finally, the population attributable risk fraction (PARF) of poverty was estimated (Details in the Methods section). The PARF calculates the possible reduction in criminal convictions assuming successful early anti-poverty intervention in the life of all the children. In a scenario without poverty, nearly a quarter (22.5%, 95% CI [5.9–36.1%]) of criminal convictions could have been prevented (Table 2).

**Table 2:** Childhood individual and family modifiable risk factors of criminal conviction.

Risk Factors	Bivariate		Adjusted^a^		PARF^c^ (99.8% CI)
	OR (95% CI)	*P* value	OR (99.8% CI)	*P* value^b^	
**Perinatal**					
Unplanned pregnancy	1.52 (0.77–3.00)	0.226	1.93 (0.52–7.26)	0.122	
Adolescent motherhood	2.50 (1.12–5.56)	0.025	2.26 (0.60–8.46)	0.056	
Smoking during pregnancy	1.34 (0.67–2.68)	0.411	1.32 (0.36–4.80)	0.510	
Alcohol consumption during pregnancy	1.02 (0.52–2.02)	0.955	1.12 (0.34–3.67)	0.762	
Preterm childbirth	0.42 (0.16–1.12)	0.084	0.50 (0.10–2.50)	0.183	
Birth weight	1.24 (0.95–1.63)	0.117	1.02 (0.66–1.58)	0.902	
**Early childhood**					
Exclusive breastfeeding duration	1.07 (0.99–1.15)	0.082	1.03 (0.92–1.17)	0.382	
No childcare attendance	1.64 (0.91–2.95)	0.100	1.32 (0.45–3.91)	0.431	
**Childhood (baseline)**					
Poverty	4.91 (2.59–9.31)	< 0.001	**4.14 (1.38–12.46)**	** < 0.001**	**22.5 (5.9–36.1)**
No contact with father/deceased	1.83 (1.00–3.34)	0.049	1.81 (0.63–5.22)	0.084	
Maternal psychiatric diagnosis	0.82 (0.43–1.54)	0.530	1.03 (0.37–2.82)	0.937	
Child: Any diagnosis	2.39 (1.31–4.35)	0.004	2.15 (0.77–5.98)	0.021	
Externalizing diagnosis	3.26 (1.70–6.26)	< 0.001	2.46 (0.76–7.95)	0.018	
Internalizing diagnosis	2.07 (0.94–4.57)	0.072	2.32 (0.62–8.76)	0.050	
Family cohesion score	0.96 (0.86–1.07)	0.469	0.97 (0.80–1.17)	0.590	
Family conflict score	1.18 (1.04–1.34)	0.008	1.14 (0.91–1.44)	0.068	
Family control score	0.93 (0.79–1.09)	0.357	0.93 (0.70–1.22)	0.402	
High maltreatment	1.78 (0.91–3.51)	0.093	2.04 (0.68–6.12)	0.045	
Bullying No	1		1		
Victim	0.89 (0.40–1.96)	0.766	0.88 (0.23–3.45)	0.778	
Perpetrator	3.25 (1.23–8.56)	0.017	3.33 (0.66–16.84)	0.022	
Both	1.93 (0.78–4.78)	0.154	1.64 (0.32–8.54)	0.353	
**Academic performance**					
Below average	4.55 (2.43–8.52)	< 0.001	2.92 (0.86–9.99**)**	0.007	
Average/above average	1		1		
School dropout	1.76 (0.39–8.03)	0.463	3.18 (0.29–34.40)	0.133	
School failure	2.68 (1.47–4.89)	0.001	1.95 (0.50–7.59)	0.130	

^a^The association between each factor and crime was adjusted by sex, age, city, ethnicity, and intelligence quotient.

^b^*P* values were considered significant with a conservative Bonferroni-corrected significance threshold of 0.05 divided by 24 tests = 0.002.

^c^PARF = population attributable risk fraction is the proportional reduction in crime that might be eliminated if exposure to the risk factor were reduced to an alternative ideal scenario of non-poverty.

Sensitivity analyses yielded similar results. Poverty was the only significant predictor in the: (1) analysis that excluded participants (n = 30) with conduct disorder at baseline (Supplementary Table 3); (2) subgroup analysis among male participants (Supplementary Table 4); (3) models using false discovery rate (FDR) method to adjust *P* values (Supplementary Table 5); (4) analysis that removed IPWs (Supplementary Table 6); (5) multilevel analysis including the random effect of the districts where the participants resided at baseline (Supplementary Table 7); and (6) multilevel models including the random effect of the schools where the children were recruited (Supplementary Table 8).

## Discussion

This study investigated a broad array of perinatal and childhood risk factors, measured at individual and family levels, for juvenile criminal conviction among a community-based cohort of Brazilian children and adolescents assessed at baseline (mean age = 10 years) and after 7 years. Although the majority of those who were poor at baseline did not present with a criminal conviction at follow-up, poverty during childhood was the only risk factor significantly associated with later criminal conviction. Specifically, poverty at baseline significantly contributed to nearly a quarter of criminal convictions.

Aligning with a meta-analysis^15^ showing no significant effects of distal exposures on criminal conviction, the current analyses found no association between perinatal exposures (i.e., unplanned pregnancy, prenatal smoking and alcohol exposure, prematurity, birthweight, and breastfeeding) and criminal conviction. The findings of the present study nominate a contextual childhood risk factor, poverty, as a better predictor of a criminal conviction than perinatal risk factors. Unlike previous investigations, criminal conviction was not associated with externalizing problems^19^, maternal psychiatric diagnosis^30^, lower family control^15^ or child maltreatment^11^. The results were consistent in sensitivity analysis. These findings highlight the importance of poverty in criminal conviction, over other clinical and family characteristics observed in previous studies in LMICs^15^ and HICs^11^. As most previous studies were conducted in HICs, these findings, showing a stronger association between poverty and criminal conviction than with other exposures, provide support for theories^31^ that indicate lesser influence of individual risk factors in LMICs compared to HICs, as higher social hardship in LMICs would supersede the impact of individual risk factors on criminal conviction^15^. The high PARF of poverty on criminal convictions may also be caused by the measure of poverty employed. Using a comprehensive measure of poverty, involving housing, education, wealth, and sanity deprivations; this study found a stronger association between criminal conviction and poverty than previous studies that investigated the association between low income at birth and criminal conviction among 1982^21^ and 1993^17^ Pelotas Birth Cohort participants. These findings highlight the importance of poverty in criminal conviction, owing to being a proxy to the exposure to several other adversities. Nevertheless, the present investigation did not explore the mechanisms linking poverty and criminal conviction. Previous studies^32^ have shown that poverty is related to crime via higher exposure to criminogenic settings, as greater unsupervised time spent with peers in activities that lack any goal direction. There are also studies suggesting that the effect of socioeconomic disadvantage on delinquency would be mediated by poor childrearing practices^33^ such as parental punishment and poorer quality of parental attachment^34^. However, our results do not subscribe to these pathways, because no association between family environment (parental control, conflict, and cohesion) and criminal conviction was found.

Community or societal risk factors that could help explain the association between poverty and criminal conviction, such as inequality in income levels, were not explored in the present study. High levels of income inequality are common in the cities where the study was carried out^35^, and this is a risk factor that has been associated with crime in LMICs^36^. Indeed, it is possible that the measure of poverty used may be a proxy for income inequality (e.g., those in poverty would be more likely to live in neighborhoods with high income inequality^37^), but further studies using measures at both the individual and contextual levels are needed to further understand the complexity of the association between poverty and criminal conviction in LMICs. One additional possible explanation for the association between poverty and criminal conviction is the inequity in access to effective legal support between the wealthiest and poorest families in LMICs^38^. In Brazil, for instance, the poorest families rely on free or state-funded legal assistance that is usually overloaded^39^, while wealthier families can afford exclusive attorney services. This could lead to higher conviction rates among youth from poor households. Further studies in this direction could provide recommendations for equal access to justice via efficient state-funded legal assistance for all citizens.

These findings should be interpreted with caution due to the following limitations. First, criminal convictions were assessed using self- and parental reports rather than official records, which may cause an underestimation of the main outcome. However, previous studies in Brazil show a strong association between self-reports and official crime records^19^. Additionally, to minimize the likelihood of underreporting, criminal conviction was assessed through different questions posed to youths and parents regarding criminal records and use of juvenile detention or probation services. Second, perinatal and early life risk factors were assessed retrospectively at baseline, increasing the likelihood of recall bias. Third, the PARF approach assumes causality. Even though we adjusted for covariates, potential unmeasured confounding factors (such as parental criminal involvement) could undermine the magnitude of the PARF for poverty in relation to criminal convictions. Fourth, though the focus of this work was individual and family risk factors, all these factors interact with contextual factors (school quality^40^, neighborhood indicators such as availability of sporting activities in the neighborhood^41^, criminality levels, etc.) that were not assessed in the present study. The sensitivity analyses with multilevel models were performed to consider the random effect of contextual factors at the district or school level and significant intraclass correlations suggested that crime varies according to the place where the children grew up and studied; however, exposure to poverty remained as the most robust contributor to criminal conviction later in life. Ecological evaluations including both contextual and personal/family risk factors in future studies may contribute to further understanding potential pathways for prevention at both contextual and individual levels. Finally, further longitudinal studies in LMICs are needed to test the robustness of our findings and to investigate the mechanisms underlying the link between poverty and crime.

## Conclusions

This study provides the first longitudinal evaluation of multiple perinatal, psychological, family, and school-related childhood exposures associated with youth criminal conviction in an LMIC. The findings highlight the association between poverty and criminal conviction, probably because the indicator of poverty used (education, housing, sanity, and goods deprivations) captured several disadvantages that youth growing up in poverty often face. The findings suggest that interventions during childhood which address poverty and the inherent social and economic adversity faced by children living in poverty may reduce youth criminal conviction. Specifically, effective anti-poverty interventions in childhood could reduce nearly a quarter of future youth criminal conviction. Therefore, investigating whether comprehensive childhood anti-poverty interventions including education, monetary, housing. and sanitary components may reduce criminality among young people in Brazil, will be prudent.

## Methods

### Participants

Data were retrieved from the BHRCS, a prospective longitudinal database comprising a randomly selected school-based community sample from the population and a high‐risk sub‐sample based on family history of psychopathology, in São Paulo and Porto Alegre, Brazil (recruitment was between 2009 and 2010). São Paulo is the most populated city in Brazil (11,253,503 inhabitants in 2010) and Porto Alegre is the capital of the southernmost state of the country (1,409,351 inhabitants in 2010)^42^. As some BHRCS studies require neuroimaging and laboratory data collection, the study area at recruitment included only public schools with more than 10,000 students that were close to research centers. Further details on sampling procedures and the map of the study area are included in the methodological paper of the BHRCS^29^. A description of the sampling procedures is provided in the Supplementary Text. Information collected at baseline (children aged 5–14 years, 2010–2011, n = 2511) and at a 7-year follow-up when individuals were 13–21 years of age (n = 1905, 76% retention rate) were analyzed.

All research was performed in accordance with the Declaration of Helsinki. All procedures were approved by the Ethics Committee of the Federal University of São Paulo and Hospital de Clínicas de Porto Alegre. Children’s assent and informed consent of the parents were obtained from participants.

### Materials

#### Outcome

##### Criminal convictions at the 7-year follow-up

In Brazil, full criminal responsibility is recognized from the age of 18 years^43^. Adolescent offenders (aged 12–17 years) receive a court order to comply with probatory socio-educational measures (referred to as the Assisted Freedom Program)^44^ or are admitted in a socio-educational center for adolescents^44^. “Any criminal conviction” was considered as a positive answer provided by parents/caregivers or youth to any of the following questions: *Has the youth ever used services or received support from a probation officer or court counselor?*, *Has the youth ever stayed overnight in a juvenile detention center, prison, or jail?* (These questions are part of the Service Assessment for Children and Adolescents^45^), and *Has the youth/Have you ever been convicted of a crime?* (Question included in the sociodemographic assessment). Thus, to compensate the unavailability of official records, multiple informants and different questions in the protocol were used to avoid underreporting of criminal conviction.

### Exposures

#### Perinatal characteristics (caregivers report)

Unplanned pregnancy, adolescent motherhood (< 18 years), any tobacco use during pregnancy, any alcohol consumption during pregnancy, prematurity (< 37 weeks), and birth weight (grams) were considered.

#### Early life exposures (caregivers report)

Exclusive breastfeeding duration (months) and childcare attendance (yes/no).

#### Childhood characteristics (baseline)

##### Poverty

A standardized questionnaire of the Brazilian Association of Research Companies^46^ was administered that classified families into socioeconomic groups based on the educational level of the head of the household (from “no education” to “university”), assets (e.g., number of refrigerators, computers, bathrooms), and access to public utility services (running water and paved streets). Scores ranged between 0 and 46. As the 2010 Brazilian criteria thresholds^46^ considered households with scores ≤ 13, as the poorest strata of the population; cohort participants with total scores ≤ 13 were classified as “poor.”

##### Contact with biological father

Caregivers were asked whether the biological father of the child was known and whether they were in contact with the biological father at the time of the interview. Answers to these questions were categorized as: in contact with father, no contact with father (including unknown father), and deceased father.

##### Maternal psychiatric diagnosis

The presence of any current psychiatric condition was evaluated using the Mini International Psychiatric Interview Plus^47^.

##### Child psychiatric diagnosis

The respondents were administered the Brazilian-Portuguese version of the Development and Well-being Assessment (DAWBA)^48,49^, based on caregiver reports. Psychiatric diagnoses were categorized as any disorder, internalizing disorders (including major depressive disorder, generalized anxiety disorder, obsessive–compulsive disorder, tic disorders, eating disorders, panic disorder, agoraphobia, social anxiety, specific phobias, and separation anxiety) and externalizing disorders (including conduct, oppositional defiant, and attention deficit/hyperactivity disorders).

##### Family cohesion, conflict, and control

The subscales of the Family Environment Scale (FES)^50^ evaluated parent/caregiver’s agreement with statements illustrating family dynamics through “true” or “false” responses. Family cohesion (example: “Your family members really help and support each other”), conflict (“You fight a lot in the family”) and control (“There are few rules to follow in your family”) subscales comprise nine, ten, and eight items respectively. Sub-scores were computed by summing items within specific dimensions. Scores ranged between 0 and 10, where higher scores indicated greater cohesion, conflict, and control. The Portuguese version of the FES demonstrates acceptable psychometric properties^51^.

##### Child maltreatment

Children and their caregivers answered questions about physical abuse (“seriously beaten by an adult at home, hurting them, or leaving bruises or marks”), physical neglect (“not enough to eat” or “forced to use dirty or torn clothes”), emotional abuse (“abused with words like *stupid*, *idiot*, *dumb*, or *useless*” or “exposed to someone shouting or screaming”) and sexual abuse (as reported by caregivers: “Has anyone ever sexually exploited the child” or “threatened to hurt them if the child refused to comply?”)^52^. Responses were rated on a 4-point scale: 0 = never; 1 = one or two times; 2 = sometimes; 3 = frequently. Based on previous psychometrics results^52^, levels of maltreatment exposure were classified as high or low. High exposure was defined as physical abuse rated ≥ 2, physical neglect and sexual abuse rated ≥ 1, and emotional abuse rated 3^52^.

##### Bullying perpetration and victimization

Caregivers received the following explanation: “We consider that a person is bullied when a student or group of students says or does unpleasant and mean things to them. Bullying also includes repeated harassment. Examples of bullying include giving nasty nicknames; humiliating, assaulting, or hurting a helpless peer; pushing; breaking and/or stealing belongings; chasing; isolating; ignoring; causing distress; etc.” Caregivers’ responses to the questions: “Has the child ever been bullied?” and “Did the child ever bully someone?” were categorized as: no bullying, bullying victim, bullying perpetrator, and bullying victim and perpetrator.

##### Academic performance

Using the Brazilian version of the Child Behaviour Checklist for 6–18 years^53^, caregivers qualified their child’s academic performance for the following subjects: Portuguese or Literature, History or Social Studies, English or Spanish, Mathematics, Biology, Sciences, Geography, and Computer Studies, as average (1), above average (2) and below average (0) compared with their peers. Z-scores derived from a previous confirmatory factor analysis^54^ were used and classified individuals as average, above average (> 1SD), and below average (< 1SD) in their academic performance.

##### Lifetime school dropout and school failure

Reported by parents/caregivers at baseline.

### Covariates

Based on previous research^33^, age, sex, ethnicity (self-reported by caregivers as White, Black, Asian, Indigenous, or Mixed-race), city, and intelligence quotient (IQ) were selected as unmodifiable covariates that could be associated with criminal conviction. IQ was assessed at baseline by trained psychologists using the vocabulary and block design subtests of the Wechsler Intelligence Scale for Children, 3rd edition – WISC-III^55^. Brazilian norms were applied^56^.

### Data analysis

All analyses were conducted using Stata version 16^57^. Sampling weights depending on sample selection (community or at high-risk, as detailed in the Online-only text)^58^ and attrition were applied in all analyses. IPWs were used to handle attrition bias as this method ensures compatibility between original and final sample^59^. Briefly, logistic regression models identified predictors of attrition based on all study variables collected at baseline. The predicted probabilities of losses according to significant covariates were used to estimate propensity scores. The IPWs were generated by weighing complete cases by the inverse of their propensity of being a complete case (Supplementary Table 2)^59^.

First, the bivariate association between criminal conviction and each one of the 22 modifiable risk factors under study was estimated using logistic regression models. Multivariable models were then estimated. In these models, each modifiable risk factor was adjusted by predefined covariates: sex, age, IQ, and ethnicity. As there were few Asian (n = 0 in the group of criminal conviction) and Indigenous (n = 1 in the group of criminal conviction) participants, ethnicity was recoded as White or Non-white. To minimize the likelihood of type I error, considering that 24 statistical tests were performed (because one of the 22 risk factors, bullying, had four categories), *P* values were adjusted using a conservative Bonferroni-corrected significance threshold. As a result, a *P* = 0.002 (alpha = 0.05/24 tests) and a 99.8% confidence interval (CI) were adopted as parameters for statistical significance for multivariable analyses.

Finally, the PARF for criminal conviction related to significant modifiable risk factors at baseline were calculated. The PARF represents the proportion of crime in the total population attributable to each predictor^60^. This helps estimate the proportion of criminal convictions which are preventable by successfully addressing the risk factors^60^. PARF was estimated after fitting the multivariable logistic regression model that included poverty as predictor using the Stata’s *punaf* command^61^. This command calculates the PARFs based on the predicted prevalence ratio estimated from two scenarios, an ideal scenario assuming all cohort participants had no exposure to poverty at baseline, divided by the prevalence in one scenario using observed data (where the risk factor of poverty is present). This ratio is known as the population unattributable fraction (PUF). Finally, *punaf* subtracts the PUF (and its confidence intervals) from 1 to obtain the PARF and its confidence intervals^60^.

Six sensitivity analyses for multivariable models were performed. First, to ensure that risk factors of incident crime were evaluated, individuals with a diagnosis of conduct disorder at baseline (n = 30) were excluded from the analyses. Second, to ensure that significant associations were not overlooked in the overall analysis, a subgroup analysis among male participants was performed. Third, an alternative *P* value adjustment using the FDR method was also computed. Fourth, the results without IPWs are also presented. The two latter sensitivity analyses were multilevel logistic regression models. These models estimated the fixed effect of each potential risk factor while adjusting for the random variation in criminal conviction according to the district of residence (fifth sensitivity analysis) or the school where the children were recruited (sixth sensitivity analysis). The procedures to perform and evaluate the results of both multilevel models involved 1) the estimation of null models including only the outcome and the random-effect level variable (district or school); 2) the evaluation of the intraclass correlation and its confidence intervals in these null models; 3) the inclusion of the main predictor (poverty) and covariates; and 4) the evaluation of model fit indices through the log-pseudolikelihood, Akaike Information Criteria (AIC), and Bayesian Information Criteria (BIC), where lower values represent better fit to the data.

### Ethics declarations

All research was performed in accordance with the Declaration of Helsinki. All procedures were approved by the Ethics Committee of the Federal University of São Paulo and Hospital de Clínicas de Porto Alegre. Child assent and parental informed consent were obtained from all the research subjects.

## Supplementary Information

Supplementary Information.

## Data Availability

CZ have full access to all the data used in the study and takes responsibility for the integrity of the data and the accuracy of the data analysis. Data were provided by the Brazilian High-Risk Cohort study and are available upon request in the Open Science Framework public repository (https://osf.io/ktz5h/).
