# Supplementary material for: Childhood individual and family modifiable risk factors for criminal conviction: a 7-year cohort study from Brazil
Source: Sci Rep. 2022 Aug 4;12:13381. doi: 10.1038/s41598-022-13975-8 (PMC9352677; doi:10.1038/s41598-022-13975-8)
Supplement: Supplementary file 1 — Supplementary Information. [file 41598_2022_13975_MOESM1_ESM.pdf]

Supplementary Information

Childhood individual and family modifiable risk factors for criminal conviction: A seven-year cohort study from Brazil

Carolina Ziebold, Sara Evans-Lacko, Mário César Rezende Andrade, Maurício Scopel Hoffmann, Lais Fonseca, Matheus Ghossain Barbosa, Pedro Mario Pan, Euripedes Miguel, Rodrigo Affonseca Bressan, Luis Augusto Rohde, Giovanni Abrahão Salum, Jair de Jesus Mari, Ary Gadelha

Supplementary Text

The Brazilian High-Risk Cohort Study for Psychiatric Conditions (BHRCS) is a school-based community cohort enriched for high family risk for psychiatric conditions (Salum et al, 2015). The study presented a two-stage design, with screening and assessment phases. At screening phase, on compulsory school registration days in 2010, all parents at public schools (22 schools in Porto Alegre and 35 in São Paulo) were invited to participate. Of 12,500 approached, 8,012 caregivers (87.3% mothers) of 9,937 eligible children aged 5-12 agreed to be screened with a modified version of the Family History Screen (FHS) by lay interviewers (Weissman et al., 2000). The FHS is a structured interview used to screen all family members for psychiatric conditions based on DSM-IV criteria. The version used had 29 main questions plus 19 conditional questions on main psychiatric syndromes. From these syndromes, attention deficit/hyperactivity disorder, anxiety disorders, obsessive-compulsive disorder, psychotic-like symptoms, learning and language problems and the child's number of symptoms of the same conditions were used in a risk prioritisation procedure. Based on the percentage of members in the family that screened positively for each of the disorders assessed, we created a family liability index adjusted for relatedness. Therefore, among the 9,937 eligible children, we recruited two subgroups: a random subsample (n=957) and a high-risk sub-sample (n=1,554) based on the family liability index. The purpose of this procedure is to observe higher incidence of psychiatric conditions over time. These subjects (N=2,511) were selected for full household assessment phase by lay interviewers (parent interview) and trained psychologists (subject's interview) at baseline (5 to 14 years).

References

Salum GA, Gadelha A, Pan PM, et al. High risk cohort study for psychiatric disorders in childhood: rationale, design, methods and preliminary results. *Int J Methods Psychiatr Res*. 2015;24(1):58-73. doi:10.1002/mpr.1459

Weissman MM, Wickramaratne P, Adams P, Wolk S, Verdeli H, Olfson M. Brief screening for family psychiatric history: the family history screen. Arch Gen Psychiatry. 2000 Jul;57(7):675-82. doi: 10.1001/archpsyc.57.7.675

Supplementary Table 1. Longitudinal studies on modifiables childhood risk factors of criminal convictions in low- and middle-income countries.

Study name and country	Design and participants	Childhood exposures	Outcome	Data analysis	Main Findings
Prague study, Czech Republic<br><br>Kubička *et al* 1995[1]<br>David, 2006[2]	Cohort of 220 subjects born to mothers who in 1961-63 were refused abortion<br>and 220 matched controls	Unwanted pregnancy	Official registers on prison sentences were retrieved at 21-23, 28, 30-31, and 35 years.	Logistic regression models adjusted by gender.	Unwanted pregnancy subjects had a double prevalence of prison sentences than controls at age 21-23[1]. No significant association at older ages[2].
Quatre Bornes and Vacoas Birth cohorts, Mauritius<br><br>Gao *et al* 2010[3]	All children born in 1969 and 1970 in two towns (Quatre Bornes and Vacoas) were recruited at age 3 years (n=1,795). Participants of this study were 137 cohort members with criminal records and 274 matched on age, gender, ethnicity, and social adversity index score controls	Electrodermal fear conditioning at age 3	Official court records of offenses: property, drug, violence, and serious driving offenses at 23 years	Repeated-measures analysis of variance	Criminal offender group failed to show fear conditioning at age 3 compared to the controls (F=4.554, df=1, 409, p=0.033)
Gao *et al* 2013[4]	73 criminals and 123 noncriminal controls at age of 23.	Reduced P3 amplitude (information-processing deficit) at age 11.<br><br>Antisocial behaviour and hyperactivity at age 11.		P3 amplitude: ANOVA and ANCOVA analysis including antisocial behaviour at age 11 and alcohol use at age 23 as covariates<br>Antisocial behaviour and hyperactivity: T-tests	Criminal offenders had significantly smaller P3 amplitudes than the controls (ANOVA= p = .03 d = 0.32), ANCOVA p = .048, η2 = 0.028)<br>Criminals and controls did not differ on age 11 antisocial

					behavior, and hyperactivity.
1982 Pelotas Birth Cohort, Brazil<br><br>Caicedo *et al* 2010 [5]	Birth cohort of 5914 livebirths in the urban area of the Pelotas city (middle-size city in Southern Brazil) in 1982.	Variables collected at childbirth:<br>Mother's skin colour<br>Maternal age <20<br>Single mother<br>Low family income (<1 minimum wages)<br>Smoking during pregnancy<br>Obstetric complications<br>Low birthweight<br>Collected at 1, 2 and 4 years:<br>Duration of partial breastfeeding<br>Duration of predominant breast feeding<br>Collected at age of 4 years:<br>Number of younger siblings<br>Number of older siblings	City and State's official records on criminal conviction due to a violent act between ages 12 and 25 years (available for 5228 participants)	Multivariable Poisson regression analyses stratified by gender.	Increased the incidence of criminal conviction at the age of 25 years: Non-white maternal skin color (females CIR [cumulative incidence ratio]=2.3, 95%CI=1.08, 5.2; males CIR=1.8, 95%CI=1.1, 2.9) and lower income at birth (males CIR=10.6, 95%CI=1.4, 78.8; females CIR=9.2, 95%CI=1.1, 74.4). Adolescent mother increased the risk only among females (CIR=2.9, 95%CI=1.3, 6.4), and having one younger sibling was associated with criminal conviction only among males (CIR=1.9, 95%CI=1.2, 3.0).

1993 Pelotas Birth cohort, Brazil<br><br>Murray *et al* 2015a[6]	Birth cohort of 5249 livebirths in the urban area of the Pelotas city in 1993.	Perinatal exposures: unplanned pregnancy, smoking in pregnancy, alcohol use in pregnancy, maternal urinary infection during pregnancy, intrauterine growth restriction; and premature birth. Cumulative number of perinatal risk factors was summed (0-6). Sociodemographic risk factors: maternal age low maternal education; single mother, three or more siblings; lowest quintile of income. Cumulative sociodemographic risk factors 0-5.	Self-reported crimes committed in the previous 12 months and official records on criminal conviction between 16 and 18 years (n=3618)<br>The association between self-reported violence and official record of violent crime at age 18 was strong (risk ratio = 5.2).	Risk ratios were computed (not clear if only bivariate analyses are presented).	Unplanned pregnancy (RR=1.5, 95%CI=1.1–2.1, mother smoked in pregnancy (RR=1.7, 95%CI=1.2–2.2) and low maternal education (RR=1.4, 95%CI=1.0–1.9) were associated with crime among females. Alcohol use in pregnancy (RR=1.5, 95%CI=1.1–2.1) was the unique predictor of crime among males. The cumulative number of perinatal (RR=1.2, 95%CI=1.1–1.4) and sociodemographic (RR=1.2, 95%CI=1.1–1.4) risk factors were associated with crime only among females.
Murray *et al* 2015b[7]		Conduct problems and hyperactivity (Strengths and Difficulties Questionnaire) at age 11	Only self-reported crime was considered. Nonviolent crimes included any of the following= stole from shops/stores, damaged property, stole from vehicle, stole vehicle, sold drug, burgled, sold	Multivariable Poisson regression models with robust standard errors. Adjusted by unplanned pregnancy, smoking during pregnancy, alcohol use in pregnancy, maternal	Conduct problems at age 11 predicted both non-violent and violent crime at age of 18 among both genders (females RR=2.4, CI95% 1.3–4.3; males RR=1.4 95%CI=1.0–2.0).

			stolen good, arson, stole from person without threat/force; and violent crime included any positive response to: stole from person with threat/force, assault, carried a weapon for fights or self-defence, used weapon.	age, number of siblings, maternal education and family income in perinatal period; parental crime birth-age 11, and child age in months at time of crime assessment.	Hyperactivity at age of 11 only predicted violent crimes for both genders (female RR=1.8, 95%CI=1.3–2.5; males RR 1.3, 95%CI=1.1–1.6).
Murray *et al* 2016[8]		Heart rate at age of 11[8a]	Self-reported crimes committed in the previous 12 months and official records on criminal conviction between 16 and 18 years (n=3613)	Multivariate logistic regression models, stratified by gender and adjusted by unplanned pregnancy, smoking during pregnancy, alcohol use in pregnancy, maternal age, number of siblings, maternal education and family income in perinatal period; child skin colour, smoking, drinking, physical activity, height, weight, blood pressure; mother's mental health.	Lower heart rate at age of 11 predicted crimes at age of 18 only among males (OR=1.46, 95%CI=1.01–1.65).[8]

[a]Heart rate were also collected at 15 and 18 years, but would not be considered childhood exposure

References

1. Kubička, L. *et al.* Children from unwanted pregnancies in Prague, Czech Republic revisited at age thirty. *Acta Psychiatr. Scand.* **91**, 361–369 (1995).

2. David, H. P. Born Unwanted, 35 Years Later: The Prague Study. *Reprod. Health Matters* **14**, 181–190 (2006).

3. Gao, Y., Raine, A., Venables, P. H., Dawson, M. E. & Mednick, S. A. Association of Poor Childhood Fear Conditioning and Adult Crime. *Am. J. Psychiatry* **167**, 56–60 (2010).

4. Gao, Y., Raine, A., Venables, P. H. & Mednick, S. A. The Association Between P3 Amplitude at Age 11 and Criminal Offending at Age 23. *J. Clin. Child Adolesc. Psychol.* **42**, 120–130 (2013).

5. Caicedo, B., Gonçalves, H., González, D. A. & Victora, C. G. Violent delinquency in a Brazilian birth cohort: the roles of breast feeding, early poverty and demographic factors. *Paediatr. Perinat. Epidemiol.* **24**, 12–23 (2010).

6. Murray, J. *et al.* Perinatal and sociodemographic factors at birth predicting conduct problems and violence to age 18 years: comparison of Brazilian and British birth cohorts. *J. Child Psychol. Psychiatry* **56**, 914–922 (2015).

7. Murray, J. *et al.* Childhood behaviour problems predict crime and violence in late adolescence: Brazilian and British birth cohort studies. *Soc. Psychiatry Psychiatr. Epidemiol.* **50**, 579–589 (2015).

8. Murray, J. *et al.* Low resting heart rate is associated with violence in late adolescence: a prospective birth cohort study in Brazil. *Int. J. Epidemiol.* **45**, 491–500 (2016).

Supplementary Table 2. Differences between original sample and second follow-up participants (BHRC, N=2,511)

N or M (% or SD)		Missing n=606		Non-missing n=1,905		OR	*P* value Before IPSW	*P* value After IPSW
Age		10.05	(1.88)	10.25	(1.91)	**0.95**	**0.030**	0.876
Gender Male		342	(56.4	1,033	(54.2)	0.91	0.341	-
Site	Porto Alegre	253	(41.8)	1,002	(52.6)	**1.55**	**<0.001**	0.791
Skin color	White	357	(58.9)	1,162	(61.0)	Ref		-
	Black	56	(9.2)	208	(10.9)	0.88	0.416	-
	Mixed	189	(31.2)	517	(27.1)	1.19	0.096	-
	Indigenous	2	(0.3)	9	(0.5)	0.72	0.680	-
	Asian	1	(0.2)	4	(0.2)	0.81	0.854	-
Planned pregnancy		190	(31.4)	593	(31.1)	1.01	0.904	-
Adolescent mother at childbirth		55	(9.2)	168	(8.9)	1.04	0.822	-
Smoking during pregnancy		140	(23.1)	431	(22.7)	1.03	0.802	-
Alcohol consumption during pregnancy		116	(19.2)	429	(22.6)	0.81	0.077	-
Preterm childbirth		72	(12.2)	293	(15.6)	**0.75**	**0.039**	0.932
Birthweight		3212.3	(600.4)	3212.2	(586.3)	1.00	0.997	-
Exclusive breastfeeding duration		4.0	(3.6)	3.9	(3.3)	1.01	0.412	-
Childcare attendance		302	(49.8)	1,089	(57.3)	**0.74**	**0.001**	0.986
Poverty		75	(12.4)	220	(11.6)	1.08	0.582	-
No contact with father		198	(32.7)	477	(25.0)	**1.45**	**0.001**	0.752
Maternal psychiatric diagnosis		157	(25.9)	584	(30.7)	**0.79**	**0.026**	0.944
Child: Any diagnosis		129	(21.3)	523	(27.5)	**0.72**	**0.003**	0.864
Externalizing diagnosis		78	(14.3)	280	(14.7)	0.86	0.263	-
Internalizing diagnosis		60	(9.9)	285	(15.0)	**0.63**	**0.002**	0.230
Intelligence Quotient		102.5	(15.97)	101.4	(17.0)	1.00	0.198	-
Family cohesion score		7.51	(1.93)	7.49	(1.90)	1.00	0.844	-
Family conflict score		3.37	(2.34)	3.42	(2.24)	0.99	0.633	-
Family control score		4.58	(1.67)	4.59	(1.60)	1.00	0.893	-
Child maltreatment								
	Low level	413	(78.1)	1,318	(76.9)	Ref		
	High level	116	(21.9)	396	(23.1)	0.94	0.573	-
Bullying	No	333	(59.8)	1,028	(58.2)	Ref		
	Victim	130	(23.3)	435	(24.6)	0.92	0.495	-
	Perpetrator	24	(4.3)	85	(4.8)	0.87	0.566	-
	Both	70	(12.6)	218	(12.3)	0.99	0.954	-
Academic performance								
	Below average	80	(13.4)	273	(14.6)	Ref		
	Average	428	(71.8)	1,336	(71.3)	1.11	0.445	-
	Above average	88	(14.8)	265	(14.1)	1.23	0.211	-
School failure		123	(20.3)	374	(19.7)	1.04	0.733	-
School dropout		12	(2.0)	40	(2.1)	0.94	0.851	-

IPSW=Inverse propensity score weighting

Supplementary Table 3. Perinatal and childhood risk factors of criminal conviction excluding participants with conduct disorders at baseline (N=1,875)

Risk Factors	Adjusted[a] OR (99.8% CI)	*P* value[b]	PARF[c] (95% CI)
Perinatal			
Unplanned pregnancy	1.83 (0.48-6.98)	0.16	
Adolescent mother at childbirth	2.53 (0.67-9.60)	0.03	
Smoking during pregnancy	1.09 (0.25-4.68)	0.86	
Alcohol consumption during pregnancy	1.08 (0.31-3.76)	0.85	
Preterm childbirth	0.53 (0.11-2.67)	0.23	
Birth weight	1.04 (0.66-1.66)	0.79	
Early childhood			
Exclusive breastfeeding duration	1.04 (0.93-1.17)	0.26	
No childcare attendance	1.36 (0.44-4.21)	0.41	
Childhood (baseline)			
Poverty	**4.17 (1.34-13.02)**	**<0.001**	**22.4 (5.1-36.6)**
No contact with father/deceased	1.77 (0.59-5.36)	0.11	
Maternal psychiatric diagnosis	0.96 (0.33-2.81)	0.91	
Child: Any diagnosis	1.84 (0.61-5.54)	0.09	
Externalizing diagnosis	1.96 (0.52-7.39)	0.12	
Internalizing diagnosis	2.21 (0.54-9.02)	0.08	
Family cohesion score	1.01 (0.82-1.23)	0.95	
Family conflict score	1.11 (0.87-1.40)	0.18	
Family control score	0.98 (0.75-1.29)	0.82	
High maltreatment	1.69 (0.52-5.48)	0.17	
Bullying No	1		
Victim	0.72 (0.17-3.11)	0.49	
Perpetrator	2.98 (0.52-16.92)	0.05	
Both	1.64 (0.30-9.12)	0.37	
Academic performance			
Below average	3.07 (0.87-10.89)	0.01	
Average/above average	1		
School dropout	3.34 (0.30-36.74)	0.12	
School failure	1.62 (0.38-6.93)	0.30	

[a] The association between each factor and crime was adjusted by sex, age, city, ethnicity, and intelligence quotient.
[b] P-values were considered significant with a conservative Bonferroni-corrected significance threshold of 0.05 divided by 24 tests= 0.002.
[c] PARF=population attributable risk fraction is the proportional reduction in crime that might be eliminated if exposure to the risk factor were reduced to an alternative ideal scenario of non-poverty

Supplementary Table 4. Perinatal and childhood risk factors of criminal conviction among males (N=1,033)

Risk Factors	Adjusted[a] OR (99.8% CI)	*P* value[b]	PARF[c] (95% CI)
Perinatal			
Unplanned pregnancy	2.21 (0.46-10.70)	0.12	
Adolescent mother at childbirth	3.09 (0.78-12.35)	0.01	
Smoking during pregnancy	1.05 (0.20-5.49)	0.93	
Alcohol consumption during pregnancy	1.08 (0.26-4.39)	0.87	
Preterm childbirth	0.58 (0.10-3.42)	0.34	
Birth weight	0.93 (0.54-1.61)	0.70	
Early childhood			
Exclusive breastfeeding duration	1.00 (0.86-1.16)	0.98	
No childcare attendance	1.44 (0.39-5.26)	0.39	
Childhood (baseline)			
Poverty	**4.91 (1.41-17.08)**	**<0.001**	**25.1 (5.8-40.4)**
No contact with father/deceased	1.57 (0.46-5.38)	0.26	
Maternal psychiatric diagnosis	0.64 (0.18-2.31)	0.28	
Child: Any diagnosis	1.73 (0.53-5.61)	0.15	
Externalizing diagnosis	2.21 (0.58-8.45)	0.07	
Internalizing diagnosis	1.24 (0.17-8.91)	0.74	
Family cohesion score	0.99 (0.80-1.24)	0.94	
Family conflict score	1.09 (0.84-1.42)	0.31	
Family control score	0.92 (0.69-1.22)	0.36	
High maltreatment	1.45 (0.42-5.05)	0.36	
Bullying No	1		
Victim	0.86 (0.18-4.06)	0.76	
Perpetrator	2.53 (0.38-16.99)	0.13	
Both	1.53 (0.25-9.43)	0.47	
Academic performance			
Below average	2.14 (0.50-9.22)	0.11	
Average/above average	1		
School dropout	4.74 (0.31-72.66)	0.08	
School failure	2.65 (0.50-14.01)	0.07	

[a] The association between each factor and crime was adjusted by age, city, ethnicity, and intelligence quotient.
[b] P-values were considered significant with a conservative Bonferroni-corrected significance threshold of 0.05 divided by 24 tests= 0.002.
[c] PARF=population attributable risk fraction is the proportional reduction in crime that might be eliminated if exposure to the risk factor were reduced to an alternative ideal scenario of non-poverty

Supplementary Table 5. Multivariable analysis: False discovery rate-adjusted p values

Risk Factors	Adjusted-*P* value[a]
Perinatal	
Unplanned pregnancy	0.26
Adolescent mother at childbirth	0.18
Smoking during pregnancy	0.64
Alcohol consumption during pregnancy	0.87
Preterm childbirth	0.31
Birth weight	0.94
Early childhood	
Exclusive breastfeeding duration	0.57
No childcare attendance	0.57
Childhood (baseline)	
Poverty	**0.02**
No contact with father/deceased	0.19
Maternal psychiatric diagnosis	0.94
Child: Any diagnosis	0.12
Externalizing diagnosis	0.10
Internalizing diagnosis	0.17
Family cohesion score	0.71
Family conflict score	0.19
Family control score	0.56
High maltreatment	0.20
Bullying	
Victim	0.85
Perpetrator	0.16
Both	0.56
Academic performance	
Below average	0.12
School dropout	0.26
School failure	0.24

[a] Each risk factor was adjusted by sex, age, ethnicity, city and Intelligence Quotient. *P* values were adjusted using the False Discovery Rate method proposed by Benjamini and Hochberg (1995). We set the significance level at 5% and raw *P* values from the logistic regression models were ranked in ascending order. Each *P* value was multiplied by the number of tests computed (24) and divided by their order in the ranking. Only adjusted *P* values <.05 were considered significant.

Supplementary Table 6. Multivariable analysis: Results without inverse-propensity score weights

Risk Factors	Adjusted[a] OR (99.8% CI)	*P* value[b]	PARF[c] (95% CI)
Perinatal			
Unplanned pregnancy	1.84 (0.49-6.90)	0.15	
Adolescent mother at childbirth	2.05 (0.56-7.53)	0.09	
Smoking during pregnancy	1.32 (0.37-4.68)	0.50	
Alcohol consumption during pregnancy	1.16 (0.36-3.77)	0.70	
Preterm childbirth	0.49 (0.10-2.32)	0.15	
Birth weight	1.02 (0.69-1.58)	0.89	
Early childhood			
Exclusive breastfeeding duration	1.04 (0.92-1.17)	0.32	
No childcare attendance	1.01 (0.45-2.26)	0.97	
Childhood (baseline)			
Poverty	**4.20 (1.41-12.56)**	**<0.001**	**22.7 (6.1-36.3)**
No contact with father/deceased	1.79 (0.63-5.09)	0.08	
Maternal psychiatric diagnosis	1.08 (0.39-2.97)	0.82	
Child: Any diagnosis	2.26 (0.81-6.29)	0.01	
Externalizing diagnosis	2.50 (0.77-8.04)	0.02	
Internalizing diagnosis	2.34 (0.62-8.89)	0.05	
Family cohesion score	0.97 (0.80-1.17)	0.58	
Family conflict score	1.14 (0.91-1.43)	0.08	
Family control score	0.91 (0.69-1.21)	0.32	
High maltreatment	2.08 (0.70-6.24)	0.04	
Bullying No	1		
Victim	0.93 (0.24-3.64)	0.87	
Perpetrator	3.53 (0.70-17.89)	0.02	
Both	1.48 (0.29-7.55)	0.47	
Academic performance			
Below average	2.81 (0.84-9.43)	0.01	
Average/above average	1		
School dropout	3.08 (0.29-32.69)	0.14	
School failure	2.07 (0.53-8.05)	0.10	

[a] The association between each factor and crime was adjusted by age, city, ethnicity, and intelligence quotient.
[b] P-values were considered significant with a conservative Bonferroni-corrected significance threshold of 0.05 divided by 24 tests= 0.002.
[c] PARF=population attributable risk fraction is the proportional reduction in crime that might be eliminated if exposure to the risk factor were reduced to an alternative ideal scenario of non-poverty

Supplementary Table 7. Modifiable risk factors of criminal conviction: Multilevel analysis including the random effect of the districts where the participants resided at baseline[a]

Risk Factors	Adjusted[b] OR (99.8% CI)	*P* value[c]	PARF[d] (95% CI)
Perinatal			
Unplanned pregnancy	1.86 (0.61-5.64)	0.09	
Adolescent mother at childbirth	2.98 (0.83-10.70)	0.01	
Smoking during pregnancy	1.19 (0.33-4.34)	0.68	
Alcohol consumption during pregnancy	0.98 (0.36-2.72)	0.96	
Preterm childbirth	0.43 (0.09-2.09)	0.01	
Birth weight	1.04 (0.63-1.69)	0.83	
Early childhood			
Exclusive breastfeeding duration	1.04 (0.92-1.19)	0.32	
No childcare attendance	1.47 (0.40-5.41)	0.37	
Childhood (baseline)			
Poverty	**4.67 (1.18-18.42)**	**0.001**	**19.5 (4.8-31.9)**
No contact with father/deceased	1.98 (0.49-7.97)	0.13	
Maternal psychiatric diagnosis	1.02 (0.24-4.29)	0.97	
Child: Any diagnosis	2.20 (0.72-6.74)	0.03	
Externalizing diagnosis	2.36 (0.63-8.92)	0.05	
Internalizing diagnosis	2.42 (0.64-9.13)	0.04	
Family cohesion score	0.97 (0.76-1.23)	0.67	
Family conflict score	1.16 (0.92-1.46)	0.05	
Family control score	0.88 (0.60-1.29)	0.31	
High maltreatment	2.50 (0.86-7.24)	0.01	
Bullying No	1		
Victim	0.88 (0.22-3.48)	0.77	
Perpetrator	4.18 (0.60-28.89)	0.02	
Both	1.67 (0.30-9.16)	0.35	
Academic performance			
Below average	3.73 (0.97-14.36)	0.003	
Average/above average	1		
School dropout	2.67 (0.18-38.73)	0.26	
School failure	2.04 (0.37-11.22)	0.20	

[a] Number of Clusters: 223 districts; Intraclass correlation=0.39, 95% CI: 0.26-0.53. Model Fit information: Log pseudolikelihood = -568.84, AIC 1141.68; BIC=1152.73.

[b] The association between each factor and crime was adjusted by sex, age, city, ethnicity, and intelligence quotient.

[c] P-values were considered significant with a conservative Bonferroni-corrected significance threshold of 0.05 divided by 24 tests= 0.002.

[d] PARF=population attributable risk fraction is the proportional reduction in crime that might be eliminated if exposure to the risk factor were reduced to an alternative ideal scenario of non-poverty

Supplementary Table 8. Modifiable risk factors of criminal conviction: multilevel models including the random effect of the schools where the children were recruited [a]

Risk Factors	Adjusted[b] OR (99.8% CI)	*P* value[c]	PARF[d] (95% CI)
Perinatal			
Unplanned pregnancy	1.96 (0.74-5.18)	0.03	
Adolescent mother at childbirth	2.43 (0.65-9.07)	0.04	
Smoking during pregnancy	1.26 (0.40-3.93)	0.54	
Alcohol consumption during pregnancy	1.20 (0.41-3.49)	0.60	
Preterm childbirth	0.44 (0.09-2.24)	0.12	
Birth weight	1.07 (0.70-1.62)	0.63	
Early childhood			
Exclusive breastfeeding duration	1.04 (0.95-1.13)	0.22	
No childcare attendance	1.22 (0.33-4.45)	0.64	
Childhood (baseline)			
Poverty	**3.99 (1.40-11.37)**	**<0.001**	**19.0 (6.7-29.7)**
No contact with father/deceased	1.72 (0.59-5.02)	0.12	
Maternal psychiatric diagnosis	0.97 (0.26-3.59)	0.93	
Child: Any diagnosis	2.29 (0.71-7.40)	0.03	
Externalizing diagnosis	2.56 (0.56-11.64)	0.06	
Internalizing diagnosis	2.56 (0.52-12.55)	0.07	
Family cohesion score	0.97 (0.76-1.24)	0.72	
Family conflict score	1.16 (0.94-1.43)	0.03	
Family control score	0.91 (0.63-1.30)	0.40	
High maltreatment	2.76 (0.93-8.18)	0.004	
Bullying No	1		
Victim	0.96 (0.24-3.74)	0.92	
Perpetrator	3.46 (0.54-22.29)	0.04	
Both	1.86 (0.42-8.25)	0.20	
Academic performance			
Below average	4.11 (0.81-21.00)	0.01	
Average/above average	1		
School dropout	1.81 (0.12-27.27)	0.50	
School failure	1.84 (0.50-6.83)	0.15	

[a] Number of Clusters: 63 schools; Intraclass correlation=0.22, 95% CI: 0.12-0.37. Model Fit information: Log pseudolikelihood = -580.67, AIC=1165.34, BIC=1176.41. Children were recruited from 57 schools, however, some children changed schools between the screening phase and the interview phase of the baseline assessment. This increased the number of schools to 63.
[b] The association between each factor and crime was adjusted by sex, age, city, ethnicity, and intelligence quotient.
[c] P-values were considered significant with a conservative Bonferroni-corrected significance threshold of 0.05 divided by 24 tests= 0.002.
[d] PARF=population attributable risk fraction is the proportional reduction in crime that might be eliminated if exposure to the risk factor were reduced to an alternative ideal scenario of non-poverty
